# Resuscitation of haemorrhagic shock with normal saline vs. lactated Ringer's: effects on oxygenation, extravascular lung water and haemodynamics

**DOI:** 10.1186/cc7736

**Published:** 2009-03-04

**Authors:** Charles R Phillips, Kevin Vinecore, Daniel S Hagg, Rebecca S Sawai, Jerome A Differding, Jennifer M Watters, Martin A Schreiber

**Affiliations:** 1 Department of Medicine, Division of Pulmonary and Critical Care Medicine, Oregon Health and Science University, Physicians Pavilion, Suite 340, 3181 SW Sam Jackson Park Road, Portland, OR 97239, USA; 2 Department of Surgery, Division of Pulmonary and Critical Care Medicine, Oregon Health and Science University, Physicians Pavilion, Suite 340, 3181 SW Sam Jackson Park Road, Portland, OR 97239, USA

## Abstract

**Introduction:**

Pulmonary oedema and impairment of oxygenation are reported as common consequences of haemorrhagic shock and resuscitation (HSR). Surprisingly, there is little information in the literature examining differences in crystalloid type during the early phase of HSR regarding the development of pulmonary oedema, the impact on oxygenation and the haemodynamic response. These experiments were designed to determine if differences exist because of crystalloid fluid type in the development of oedema, the impact on oxygenation and the haemodynamic response to fluid administration in early HSR.

**Methods:**

Twenty anaesthetised swine underwent a grade V liver injury and bled without resuscitation for 30 minutes. The animals were randomised to receive, in a blinded fashion, either normal saline (NS; n = 10) or lactated Ringer's solution (LR; n = 10). They were then resuscitated with study fluid to, and maintained at, the preinjury mean arterial pressure (MAP) for 90 minutes.

**Results:**

Extravascular lung water index (EVLWI) began to increase immediately with resuscitation with both fluid types, increasing earlier and to a greater degree with NS. A 1 ml/kg increase in EVLWI from baseline occurred after administartion of (mean ± standard error of the mean) 68.6 ± 5.2 ml/kg of normal saline and 81.3 ± 8.7 ml/kg of LR (*P* = 0.027). After 150 ml/kg of fluid, EVLWI increased from 9.5 ± 0.3 ml/kg to 11.4 ± 0.3 ml/kg NS and from 9.3 ± 0.2 ml/kg to 10.8 ± 0.3 ml/kg LR (*P* = 0.035). Despite this, oxygenation was not significantly impacted (Delta partial pressure of arterial oxygen (PaO_2_)/fraction of inspired oxygen (FiO_2_) ≤ 100) until approximately 250 ml/kg of either fluid had been administered. Animals resuscitated with NS were more acidaemic (with lower lactates), pH 7.17 ± 0.03 NS vs. 7.41 ± 0.02 LR (*P* < 0.001).

**Conclusions:**

This study suggests that early resuscitation of haemorrhagic shock with NS or LR has little impact on oxygenation when resuscitation volume is less than 250 ml/kg. LR has more favourable effects than NS on EVLWI, pH and blood pressure but not on oxygenation.

## Introduction

Pulmonary oedema has been reported to be a common consequence of haemorrhagic shock and resuscitation (HSR) [1]. The early phase of resuscitation of haemorrhagic shock frequently involves the administration of crystalloid solutions prior to the arrival of blood products to attain a goal blood pressure. Lactated Ringer's solutions (LR) and normal saline (NS) remain common resuscitation fluids. Remarkably, little data exist examining the differences in these crystalloid fluids during the early resuscitation phase as a determinant of the amount of extravascular lung water (measured in terms of the extravascular lung water index (EVLWI)) formed and the impact on oxygenation [2-14]. Differences in volumes required to maintain goal mean arterial pressure (MAP) could impact both oedema formation and oxygenation but, again, remarkably little data exist examining differences in the haemodynamic response to these fluids in early HSR.

The EVLWI at any time represents a dynamic balance between factors that cause fluid to accumulate in the lungs and those that carry it out of the lungs. An increase in fluid leaving the vascular space and remaining in the pulmonary parenchyma following HSR can result from: endothelial injury with increased pulmonary capillary permeability; alveolar epithelial injury and decreased alveolar fluid clearance; dilutional decreases in serum oncotic pressure; increased pulmonary capillary pressure; coagulation abnormalities with increased extravasation of fluid; and increased inflammation [2,3]. But both lymphatic and vascular removal of EVLWI can increase by as much as 300% after haemorrhagic shock, acting to limit net EVLWI formation. Little is known about the effects of the volume and type of crystalloid administered during the early resuscitation of haemorrhagic shock on these opposing factors and their impact on the formation of clinically significant amounts of EVLWI and the impact on oxygenation.

These experiments were designed to measure the EVLWI, the partial pressure of arterial oxygen (PaO_2_)/fraction of inspired oxygen (FiO_2_) and parameters of haemodynamics during early resuscitation from haemorrhagic shock with LR or NS and determine if there are differences on the development of oedema, the impact on oxygenation and the haemodynamic response to fluid administration because of fluid type.

## Materials and methods

The study design was a randomised, blinded, controlled trial. The Institutional Animal Care and Use Committee at Oregon Health and Science University approved the protocol. This facility adheres to the National Institutes of Health guidelines for the use of laboratory animals.

Twenty Yorkshire crossbred pigs weighing approximately 35 kg underwent a 16-hour fast preoperatively with water *ad libitum*. The swine were preanaesthetised with 8 mg/kg of intramuscular tiletamine/zolazepam (Fort Dodge Animal Health, Fort Dodge, IA, USA), intubated with an oral endotracheal tube and placed on mechanical ventilation. Tidal volume was set at 12 ± 2 ml/kg, and respiratory rate was adjusted to maintain end-tidal carbon dioxide and partial pressure of arterial carbon dioxide (PaCO_2_) of 40 ± 4 mmHg. Anaesthesia was maintained using 1 to 3% isoflurane as needed. To assess adequacy of anaesthesia, we monitored jaw tone. Monitoring devices were placed after establishing anaesthesia, including an oesophageal thermometer and external jugular vein catheter. Animal temperature was maintained at 38.0 ± 1.5°C using external warming devices and warmed fluids. Femoral artery cut down was performed to place a 4-F femoral catheter with an integrated thermistor tip (Pulsion Medical Systems, Munich, Germany) for continuous blood pressure monitoring, blood sampling and transpulmonary thermodilution determinations of cardiac output (CO), EVLWI, global end-diastolic volume (GEDV) and intrathoracic blood volume (ITBV). MAP and heart rate (HR) were continuously recorded.

The transpulmonary thermodilution method using the single-indicator transpulmonary thermodilution technique (PiCCO; Pulsion Medical Systems, Munich, Germany) was originally developed in swine and has been previously validated by comparison with the postmortem gravimetric technique, and with the double dilution (thermo-dye) technique in swine, sheep and humans in a variety of disease states [15-18]. The technique can slightly overestimate EVLWI in normal controls and underestimate it in severe lung injury. However, its sensitivity has been found to allow detection of clinically relevant changes in EVLWI [19].

We examined the precision of this technique for measuring EVLWI in this species of swine using repeated measures of EVLWI at baseline in three animals in separate experiments. We found an average coefficient of variation (standard deviation/mean × 100) of less than 5%. As an example, repeated measurements (n = 20) were made in one animal at baseline yielding a mean EVLW (± standard deviation) of 9.1 ± .45 ml/kg.

For these experiments, a 15 ml bolus of iced randomised fluid (0 to 6°C) was injected via a central venous catheter into the right atrium. The thermodilution curve was recorded with a femoral artery thermistor and used to determine CO, the volume of blood in the heart at the end of filling or the GEDV, the ITBV and EVLWI, as previously described [17,20]. The average result from three consecutive 15 ml bolus injections was recorded for each animal. Knowing the GEDV and the ITBV allowed for calculation of the pulmonary blood volume (PBV). The pulmonary vascular permeability index (PVPI) was then calculated as EVLWI/PBV [21]. With increased EVLWI, a high PVPI with a low filling volume implies either greater fluid extravasation or impaired clearance of fluid from the lung or both. A lower value at the same EVLWI implies a higher central blood volume with increased hydrostatic pressures as seen in congestive heart failure. Transpulmonary thermodilution measurements were performed at baseline, immediately before injury, at the end of spontaneous bleeding, every 10 minutes during autoresuscitation, each time baseline blood pressure was attained with resuscitation or at least every 15 minutes during resuscitation, and immediately before the end of the study period. Continuous measurements of HR, blood pressure, CO (by pulse contour analysis), respiratory rate, body temperature and urine output were made throughout the entire protocol.

The animals underwent a midline coeliotomy, suprapubic urinary catheter placement and splenectomy. Splenectomies are performed in swine haemorrhage models because of the spleen's distensibility and the resultant variable amounts of sequestered blood. The spleen was weighed and randomised fluid was infused to replace three times the spleen weight. Following a 15-minute stabilisation period, a standardised grade V liver injury was created with a specially designed clamp. The clamp was directed centrally over the liver and created a consistent pattern of injury involving one or more central hepatic veins. Injuries met criteria for grade V liver injuries as described by the American Association for the Surgery of Trauma Organ Injury Scaling system [22]. This model has been described in several previous studies [23-25]. The time of injury was considered to be the start time of the two-hour study period.

After 30 minutes of uncontrolled haemorrhage, blood was evacuated from the abdomen and measured. Blinded resuscitation was begun with either LR or NS. Both fluids were purchased from Baxter (Deerfield, IL, USA) and were unmodified (NS, pH 4.5 to 7.0, 154 mEq/L sodium, 154 mEq/L chloride; LR, pH 6.0 to 7.5, 130 mEq/L sodium, 109 mEq/L chloride, 4 mEq/L potassium, 3 mEq/L calcium, 28 mEq/L l-lactate). A 30-minute delay before beginning resuscitation allowed the animals to reach their nadir blood pressure, replicating the civilian trauma scenario. Fluid was delivered at 165 mL/minute. This rate was chosen because it is one-half of the rate delivered by the level 1 infuser to humans, and the pigs were approximately one-half the weight of a normal adult human. The goal of resuscitation was to achieve and maintain the baseline MAP for 90 minutes post injury. Blood loss was determined by placing pre-weighed laparotomy sponges into the pelvis and inferior right and left pericolic gutters before creating the liver injury and collecting active haemorrhage by suction or with the sponges, while avoiding manipulation of the liver injury. The sponges were removed before abdominal closure. Following resuscitation and prior to euthanasia, the abdomen was opened and any additional blood loss was collected by suction. Blood loss (mL/kg) was reported as a mean for each resuscitation group. To ensure comparable injuries between the study groups, we removed the liver and identified the number of hepatic vessels injured.

### Statistical analysis

Comparisons between groups were made with independent-sample *t *tests using a statistical software package for personal computers (SPSS, Windows Version 11.5, SPSS, Inc., Chicago, IL, USA). Significance was defined as p < 0.05. All data are presented as means ± standard error of the mean. Linear regression analysis was performed to determine the effect of fluid type, independent of volume, as shown by differences in the slopes of the regression lines. Boxplots comparing differences in means with confidence intervals of MAP, CO, systemic vascular resistance (SVR), GEDV, stroke volume (SV) and HR were performed to examine differences in those values with NS vs. LR resuscitation.

## Results

**Figure 1:**
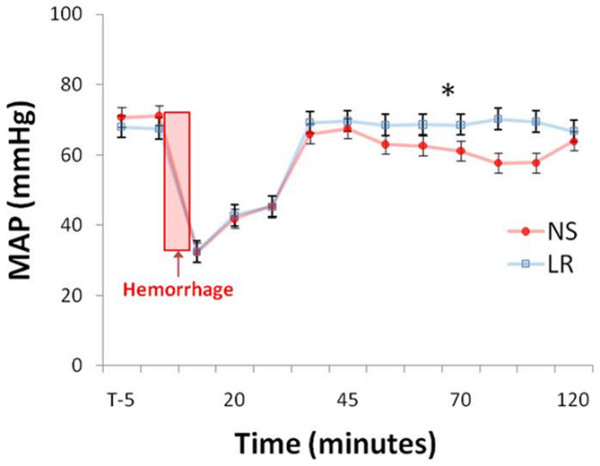
Blood pressure curves for experimental groups. This graph is a composite of the mean arterial pressures of the two resuscitation groups. The liver injury is created at time zero. Fluid resuscitation was begun at 30 minutes into the experiment. There is no difference in mean arterial pressure (MAP) between the two groups until resuscitation. Despite being given significantly more fluid, mean arterial pressures of the animals five the normal saline (NS) was significantly lower than the lactated Ringer's solution (LR) group beginning 39 minutes into the resuscitation (NS 56.9 ± 1.6 mmHg vs. LR 64.0 ± 2.0 mmHg; * p = 0.01)) and remaining so until close to study end.

One animal in the NS group did not survive the two-hour study. All the animals in the LR group survived. Composite MAP of the two groups throughout the study are shown in Figure 1. The grade V liver injury caused rapid blood loss and a rapid drop in blood pressure. On reaching a nadir blood pressure, bleeding spontaneously stopped in all animals. This was followed by a period of spontaneous increase in blood pressure that was augmented after 30 minutes with active resuscitation using either NS or LR (randomised) to baseline MAP. Despite receiving a larger resuscitation volume in an attempt to maintain target blood pressure, the average MAP during the resuscitation phase was significantly lower in the NS group (NS 56.9 ± 1.6 mmHg vs. LR 64.0 ± 2.0 mmHg, p = 0.01). Although all the animals received large resuscitations, no animal in either group developed signs of abdominal compartment syndrome such as difficulty in maintaining adequate ventilation or decreased urine output.

Mean urine output and blood loss were greater in the NS group than in the LR group. Urine output was 44.1 ml/kg ± 8.1 in the NS group vs. 19.4 ml/kg ± 3.4 ml/kg in the LR group (p = 0.012), and total blood loss was 34.3 ± 2.9 ml/kg vs. 23.7 ± 2.1 ml/kg (p = 0.009). The NS group required almost twice as much fluid (NS 330.8 ± 38.1 ml/kg vs. LR 148.4 ± 20.2 ml/kg; p = 0.009) over the 90-minute resuscitation period in an attempt to maintain goal MAP. There was no difference in blood loss prior to fluid resuscitation.

**Figure 2:**
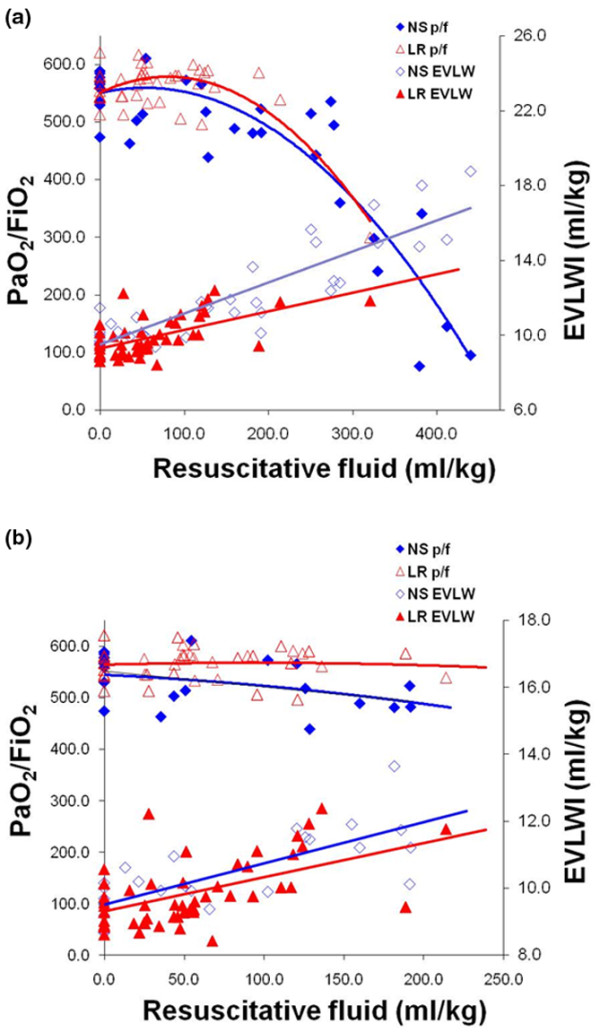
EVLWI and PaO_2_/FiO_2 _per volume of resuscitation fluid. **(a)** Extravascular lung water index (EVLWI) and partial pressure of arterial oxygen (PaO_2_)/fraction of inspired oxygen (FiO_2_) as a function of resuscitation volume over the entire study. Linear regression analysis reveals a larger increase in EVLWI in the normal saline (NS) group as compared with the lactated Ringer's solution (LR) group. An increase of 1 ml/kg of EVLWI occurred at a resuscitation volume of 55.6 ± 6.3 ml/kg for normal saline and 76.9 ± 12.1 ml/kg for LR (p = 0.02). A significant change in oxygenation defined as a drop in PaO_2_/FiO_2 _of 100 or more (roughly corresponding to a drop in arterial oxygen saturation below 90% on room air) did not occur until more than 250 ml/kg of either fluid had been administered. **(b) **EVLWI and PaO_2_/FiO_2 _as a function of resuscitation volume of 250 ml/kg or less. To examine fluid type-specific effects we examined changes in oxygenation and EVLWI at similar total volumes of resuscitation. A limit of 250 ml/kg was chosen as none of the LR animals required more than this volume to maintain goal mean arterial pressure (MAP) and to allow examination of the effects of fluid type early in the resuscitation at similar volumes. Linear regression analysis revealed a larger increase in EVLWI in the NS group as compared with the LR group at similar volumes infused as shown by differences in the slopes of the regression lines (p = 0.027). An increase of 1 ml/kg of EVLWI occurred at a resuscitation volume of 68.6 ± 5.2ml/kg for NS and 81.3 ± 8.7 ml/kg for LR (p = 0.027). A significant change in PaO_2_/FiO_2 _(≥ 100) was not seen over this range of volume.

EVLWI and the PaO_2_/FiO_2 _ratio as a function of resuscitation fluid volume over the entire study are plotted in Figure 2a. Linear regression analysis revealed a larger increase in EVLWI in the NS group as compared with the LR group (p = 0.020), with a statistically significant difference seen very early in the resuscitation. An increase of 1 ml/kg of EVLWI occurred at a resuscitation volume of 55.6 ± 6.3 ml/kg for NS vs. 76.9 ± 12.1 ml/kg for LR (p = 0.21). Although the overall increase in EVLWI was greater for the NS group as compared with the LR group there was no difference in oxygenation. A drop in PaO_2_/FiO_2 _of 100 (roughly corresponding to a drop in arterial oxygen saturation below 90% on room air) did not occur until approximately 250 ml/kg of either fluid had been administered.

Figure 2b shows EVLWI and PaO_2_/FiO_2 _plotted as a function of resuscitation fluid volume administered early in the study, limited to 250 ml/kg. This was performed to examine the effect of fluid type early in the resuscitation at similar volumes. Linear regression analysis revealed a larger increase in EVLWI in the NS group as compared with the LR group occurring early in the resuscitation. There was no significant difference in baseline EVLWI for the two groups. A 1 ml/kg increase in EVLWI from baseline occurred after 68.6 ± 5.2 ml/kg of NS and 81.3 ± 8.7 ml/kg of LR (p = 0.027). After 150 ml/kg of fluid EVLWI increased from 9.5 ± 0.3 ml/kg to 11.4 ± 0.3 ml/kg for NS and from 9.3 ± 0.2 mg/g to 10.8 ± 0.3 ml/kg for LR (p = 0.035). Significant changes in oxygenation, defined as a drop in baseline PaO_2_/FiO_2 _to 100 or greater, did not occur over this range for either fluid type.

**Figure 3:**
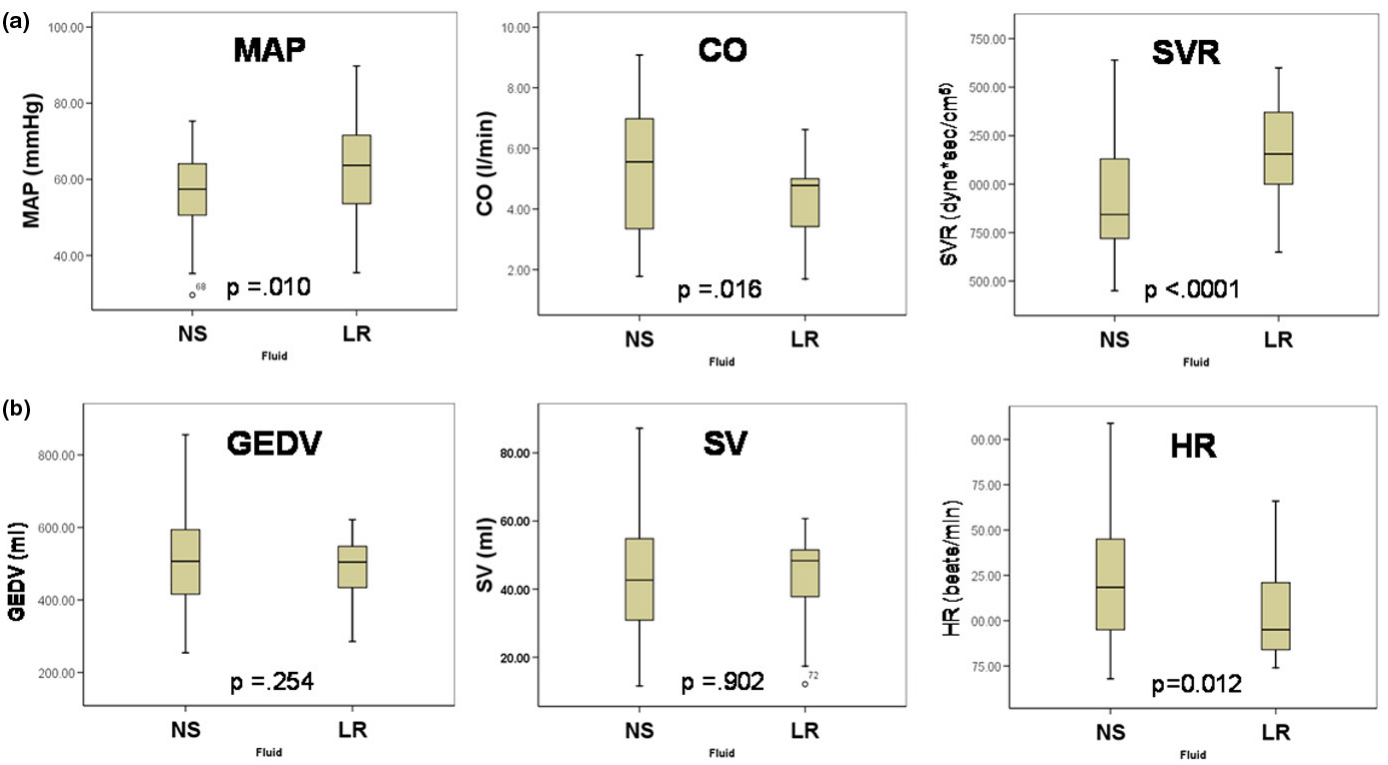
Haemodynamic data during resuscitation. Boxplots for the means of the values at the time the difference in mean arterial pressure (MAP) between the two groups first became significant. **(a)** Significant differences in MAP, cardiac output (CO) and systemic vascular resistance (SVR). There were no differences in baseline values immediately prior to resuscitation between the groups. During the resuscitation stage MAP was lower in the normal saline (NS) group (NS 56.9 ± 1.6 mmHg vs. lactated Ringer's solution (LR) 64.0 ± 2.0 mmHg; p = 0.01) despite this group having higher COs (NS 5.2 ± 0.3 l/minute vs. LR 4.4 ± 0.2 l/minute; p = 0.016). This was due to a significant difference in SVR (NS 923 ± 51.4 dyne × sec/m^3^vs. 1177.6 ± 34.6 dyne × sec/m^3^; p < 0.001). **(b)** There were no differences in the preload metric global end-diastolic volume (GEDV) or in stroke volume (SV). The differences in CO seen in (a) were due to a significant increased heart rate (HR) in the NS group (NS 114.9 ± 6.5 beats/minute vs. LR 93.6 ± 3.6 beats/minute; p = 0.012).

Figure 3a shows boxplots for the median values with confidence intervals for the average MAP, CO and SVR between the two groups during the resuscitation period at the time the difference in MAP between the two groups first became significant. There were no differences in baseline values immediately before resuscitation. The average MAP was lower in the NS group despite infusing nearly twice as much NS. CO was higher in the NS group (NS 5.2 ± 0.3 l/minute vs. LR 4.4 ± 0.2 l/minute, p = 0.016) and SVR was lower in the NS group (923 ± 51.4 dyne × sec/m^3 ^vs.1177.6 ± 34.6 dyne × sec/m^3^; p < 0.001). As shown in Figure 3b, there were no differences between groups with respect to the GEDV or the SV, and the observed difference in CO was entirely due to differences in HR (NS 114.9 ± 6.5 beats/minute vs. LR 93.6 ± 3.6 beats/minute; p = 0.012).

**Figure 4:**
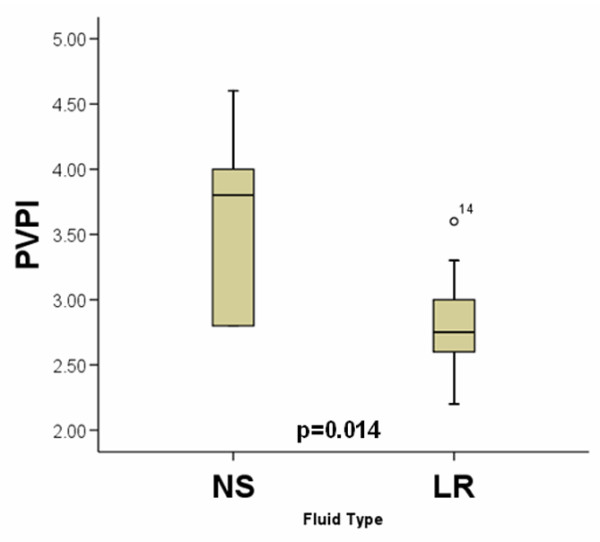
Mean PVPI during resuscitation. Boxplots for mean pulmonary vascular permeability index (PVPI) at the time when the difference in extravascular lung water index between the two groups first became significant. A significant difference between the two groups was found (normal saline (NS) 3.6 ± 0.25 vs. lactated Ringer's solution (LR) 2.9 ± 0.13; p = 0.014).

Figure 4 shows that the PVPI (defined as EVLW/PBV) obtained when the difference in EVLWI between the two groups first became significant. The PVPI was greater in the NS group (NS 3.6 ± 0.25 vs. LR 2.9 ± 0.13; p = 0.014). A higher PVPI value with the same filling volume implies either greater extravasation of fluid into the lung or an impairment of fluid clearance or a combination of the two. There was no difference in PVPI immediately before resuscitation between the two groups.

Laboratory values at the end of the study are shown in Table 1. Animals resuscitated with NS were more acidaemic (despite having lower serum lactate levels), were more anaemic and had lower serum calcium and potassium levels.

**Table 1:** Laboratory values at study end

	**Normal saline**	**Lactated Ringer's solution**	**p value**
Sodium	137.9 ± 10.8	127.0 ± 8.2	0.432
Potassium	3.5 ± 0.2	4.1 ± 0.06	0.010
Calcium	1.22 ± 0.06	1.34 ± 0.01	0.039
Lactate	1.3 ± 0.3	6.0 ± 0.7	< 0.001
Bicarbonate	16.7 ± 1.7	27.8 ± 0.9	< 0.001
Partial pressure of arterial carbon dioxide	44.7 ± 3.0	43.3 ± 0.6	0.647
pH	7.17 ± 0.03	7.41 ± 0.02	< 0.001
Haemoglobin	4.7 ± 0.39	5.9 ± 0.35	0.039
Haematocrit	12.7 ± 1.1	16.56 ± 1.2	0.028

Results are reported as means ± standard error of the mean. Animals resuscitated with normal saline were more acidaemic (despite having lower lactates), were more anaemic and had lower serum calcium and potassium levels.

## Discussion

In this swine model of traumatic haemorrhagic shock, resuscitation with NS as compared with LR resulted in greater EVLWI formation at similar amounts of resuscitation volume. In addition, the blood pressure response to fluid was considerably lower in the NS group (NS 56.9 ± 1.6 mmHg vs. LR 64.0 ± 2.0 mmHg; p = 0.01) despite requiring more total fluid over the entire study period (NS 330.8 ± 38.1 ml/kg vs. LR 148.4 ± 20.2 ml/kg; p = 0.009) in an attempt to maintain goal MAP. The lower blood pressure with NS administration was due to a greater systemic vasodilatation as compared with LR. Surprisingly neither fluid resulted in significant changes in oxygenation, as defined by a drop in baseline PaO_2_/FiO_2 _of 100 or more (roughly corresponding to a drop in arterial oxygen saturation below 90% on room air), until approximately 250 ml of either fluid had been infused. This corresponds to 17.5 litres in a 70 kg human – well in excess of what would be normally administered during most human resuscitations prior to the arrival of blood products.

We observed differences in EVLWI early in the resuscitation when similar volumes of the fluids had been given, more NS than LR. This supports the idea that a fluid specific effect, independent of volume, was present. To evaluate this we performed two separate regression analyses: Figure 2a examined the change in EVLWI as a function of fluid given over the entire study and Figure 2b examined the change in EVLWI at similar volumes of resuscitation fluid earlier in the resuscitation (< 250 ml/kg).

A second order polynomial was used as the best fit line for PaO_2_/FiO_2 _vs. volume of resuscitation for two reasons. First, previous studies have shown a stepwise increase in the rate of EVLWI formation as a function of the interstitial matrix filling and then abruptly 'giving way' with a subsequent increased rate of fluid extravasation into the matrix and then alveoli. One could expect an initial improvement in PaO_2_/FiO_2 _with improved CO early in the resuscitation, followed by a curvilinear decline as the matrix 'lets go' with a subsequent increase rate in fluid extravasation. Secondly a curvilinear line dramatically improved the regression coefficient (R^2^); from 0.620 to 0.815 for NS, and from 0.15 to 0.60 for LR. We found significant differences in EVLWI formation at similar volumes of resuscitation, more NS than LR early in the resuscitation. The cause of the greater EVLWI accumulation in the NS group at similar resuscitation volumes as compared with the LR group is not known.

EVLWI is defined as the extravascular fluid in the lung at any moment including the intracellular fluid of inflammatory, endothelial and epithelial cells in the extravascular space, as well as alveolar and interstitial fluid and intrapulmonary lymph [1]. The amount of EVLWI represents a dynamic balance between factors that cause fluid to accumulate in the lungs and those that carry it out of the lungs.

HSR results in increased expression of inflammatory cytokines, increased neutrophil sequestration in the lung, increased generation of reactive oxygen and nitrogen species, and activation of coagulation. All of these factors can cause endothelial and epithelial cell dysfunction and increased EVLWI [24,26-28]. However, our laboratory has previously shown in an identical swine model that there is no difference in the HSR-related increase in the levels of pro-inflammatory mRNA gene expression for IL-6, granulocyte colony-stimulating factor and TNF-α [24] or increased numbers of sequestered neutrophils in the lung between NS and LR resuscitated animals.

The NS group developed a non-anion gap metabolic acidosis whereas the LR group remained pH neutral. Several studies have shown potential lung protective effects of hypercapnic acidosis in acute lung injury [29-32]. However, there is a paucity of clinical data evaluating the direct effects of either hypercapnia or acidosis on EVLWI formation and the acute lung injury following haemorrhagic shock. The protective effects of hypercapnic acidosis in acute lung injury may be a function of the acidosis, the hypercapnia or a combination of both and further work is required to gain a better understanding of these effects.

Dilutional decreases in serum oncotic pressure are likely to have contributed to the rise in EVLWI seen with both fluid types in this study. It is likely that the differences in the total EVLWI measured at study end were in large part due to dilutional effects from the greater volume of NS required. Twice the volume of NS was required to maintain target MAP. This has clinical relevance in regards to selecting fluid type, in that a greater volume of NS will need to be administered to maintain blood pressure goals and will result in greater EVLWI formation and acidosis.

Figure 3 shows that there were no differences in the GEDV or SV during resuscitation between the two groups. The NS group had an increased CO as compared with the LR group, due entirely to an increased HR. We believe this occurred as a compensatory response to greater peripheral vasodilatation seen in the NS group. It is unlikely that increased preload or a decrease in cardiac function caused an increase in capillary pressures to explain the higher EVLWI in the NS animals. It remains possible that differences in capillary hydrostatic pressure existed despite not being reflected in changes in filling volumes or SV.

As shown in Figure 4 the PVPI (defined as EVLWI/PBV) was greater in the NS group (NS 3.6 ± 0.25 vs. LR 2.9 ± 0.13, p = 0.014) at a time during resuscitation when differences in EVLWI first became significant. A higher PVPI value with the same filling volume implies either greater extravasation of fluid into the lung due to increases in permeability, changes in the transcapillary oncotic pressure gradient, an impairment of fluid clearance or a combination of the three. As this occurred at a time when there were no differences in volumes of resuscitation administered or central filling volumes this finding suggests that NS may have caused a pulmonary capillary endothelial permeability injury relative to the LR group to explain the differences in EVLWI.

Total blood loss was greater in the NS group than the LR group (34.3 ± 2.9 ml/kg vs. 23.7 ± 2.1 ml/kg; p = 0.009). It is known that HSR with NS attenuates the hypercoagulable response seen with haemorrhage as compared with LR [25]. Increased bleeding during resuscitation due to a dilutional coagulopathy and an attenuation of the post-haemorrhage hypercoagable response was felt to account for the differences seen. With haemorrhage-related endothelial injury, attenuation of the hypercoagable response could also result in greater extravasation of fluid and thus explain in part the greater EVLWI seen in the NS group.

Previous studies examining the effects of NS vs. LR on EVLWI, oxygenation and the haemodynamic response have been conducted in controlled haemorrhage models. Many re-infused shed blood with the resuscitation fluid. Our study used a more clinically relevant model by adding tissue injury to uncontrolled haemorrhage and initiating early resuscitation with crystalloids alone and resuscitating to a goal blood pressure. Although this complicated the analysis of the effect of fluid type independent of volume, we felt by doing so our results could be more reliably extrapolated to the human clinical scenario. Further work is needed to examine late effects of crystalloid type and volume on EVLWI, oxygenation and haemodynamics in a living model of HSR.

Our study did have limitations. By study end the animals had received extremely large resuscitation volumes that exceeded those typically seen in human scenarios. We believe this reflects the severity of the model as well as the choice to resuscitate to the baseline MAP. This resulted in resuscitation beyond normal perfusion as evidenced by supranormal urine outputs especially in the NS group. Despite this fact, differences between the fluids manifested very early, prior to exceeding resuscitation volumes typically used clinically. We have also previously shown that differences in pH and coagulation between LR and NS occur very early after initiation of fluid resuscitation [24]. This indicates that fluid type and not only differences in fluid volume produced the observed differences in EVLWI and blood loss. We chose to not use vasoactive medications in this study, despite the large resuscitation volume, because they are currently rarely used in the early resuscitation of haemorrhagic shock.

A tidal volume of 12 ml/kg was used in this study. This tidal volume may have caused some lung injury. Although this may have played a role in the development of increased EVLWI, both animal groups were exposed to the same tidal volume making it an unlikely cause of a difference between groups.

## Conclusions

This study suggests that early resuscitation of haemorrhagic shock with NS or LR prior to the arrival of blood products is safe in terms of the immediate impact on oxygenation when resuscitation volume is limited to less than 250 ml/kg. Resuscitation with LR has more favourable effects on EVLWI formation, pH, coagulation and haemodynamics but not on oxygenation. Further work is needed to understand the cause of these fluid-specific differences.

## Key messages

• When resuscitating haemorrhagic shock prior to the arrival of blood products significantly more NS is required to maintain goal blood pressure than LR.

• LR has more favourable effects on EVLWI formation, pH, coagulation and haemodynamics than does NS.

• Further investigation is warranted to attempt to explain these fluid-specific differences.

## Abbreviations

CO: cardiac output; EVLWI: extravascular lung water index; FiO_2_: fraction of inspired oxygen; GEDV: global end-diastolic volume; HR: heart rate; HSR: haemorrhagic shock and resuscitation; IL: interleukin; ITBV: intrathoracic blood volume; LR: lactated Ringer's solution; MAP: mean arterial pressure; NS: normal saline; PaCO_2_: partial pressure of arterial carbon dioxide; PaO_2_: partial pressure of arterial oxygen; PBV: pulmonary blood volume; PVPI: pulmonary vascular permeability index; SV: stroke volume; SVR: systemic vascular resistance; TNF: tumour necrosis factor.

## Competing interests

CRP has acted as an advisor to Pulsion Medical Systems, makers of the PiCCO device that measures EVLW. There are no other potential conflicts from any of the authors.

## Authors' contributions

CRP participated in study design and coordination and helped draft the mauscript. KV helped with data collection and analysis. DSH participated in executing the experiments, and helped with data collection. RSS participated in the design of the study and participated in executing the experiments. JAD participated in the design and coordination of the study, in study execution, data collection and analysis. JMW participated in the design of the study and participated in executing the experiments. MAS conceived of the study, participated in study design and coordination and helped draft the mauscript. All authors read and approved the final manuscript.
